# Global transcript and phenotypic analysis of yeast cells expressing Ssa1, Ssa2, Ssa3 or Ssa4 as sole source of cytosolic Hsp70-Ssa chaperone activity

**DOI:** 10.1186/1471-2164-15-194

**Published:** 2014-03-14

**Authors:** Naushaba Hasin, Sarah A Cusack, Shahin S Ali, David A Fitzpatrick, Gary W Jones

**Affiliations:** Yeast Genetics Laboratory, Department of Biology, National University of Ireland Maynooth, Maynooth, County Kildare, Ireland; Section on Formation of RNA, National Institutes of Child Health and Human Development, National Institutes of Health, Bethesda, Maryland 20814 USA; United States Department of Agriculture, ARS Sustainable Perennial Crops Laboratory, Beltsville, Maryland USA; Genome Evolution Laboratory, Department of Biology, National University of Ireland Maynooth, Maynooth, County Kildare, Ireland

**Keywords:** *Saccharomyces cerevisiae*, Prion, Heat shock, Stress, Hsp70, Ssa1, Ssa2, Ssa3, Ssa4, Chaperone, Gene expression

## Abstract

**Background:**

Cytosolic Hsp70 is a ubiquitous molecular chaperone that is involved in responding to a variety of cellular stresses. A major function of Hsp70 is to prevent the aggregation of denatured proteins by binding to exposed hydrophobic regions and preventing the accumulation of amorphous aggregates. To gain further insight into the functional redundancy and specialisation of the highly homologous yeast Hsp70-Ssa family we expressed each of the individual Ssa proteins as the sole source of Hsp70 in the cell and assessed phenotypic differences in prion propagation and stress resistance. Additionally we also analysed the global gene expression patterns in yeast strains expressing individual Ssa proteins, using microarray and RT-qPCR analysis.

**Results:**

We confirm and extend previous studies demonstrating that cells expressing different Hsp70-Ssa isoforms vary in their ability to propagate the yeast [*PSI*^*+*^] prion, with Ssa3 being the most proficient. Of the four Ssa family members the heat inducible isoforms are more proficient in acquiring thermotolerance and we show a greater requirement than was previously thought, for cellular processes in addition to the traditional Hsp104 protein disaggregase machinery, in acquiring such thermotolerance. Cells expressing different Hsp70-Ssa isoforms also display differences in phenotypic response to exposure to cell wall damaging and oxidative stress agents, again with the heat inducible isoforms providing better protection than constitutive isoforms. We assessed global transcriptome profiles for cells expressing individual Hsp70-Ssa isoforms as the sole source of cytosolic Hsp70, and identified a significant difference in cellular gene expression between these strains. Differences in gene expression profiles provide a rationale for some phenotypic differences we observed in this study. We also demonstrate a high degree of correlation between microarray data and RT-qPCR analysis for a selection of genes.

**Conclusions:**

The Hsp70-Ssa family provide both redundant and variant-specific functions within the yeast cell. Yeast cells expressing individual members of the Hsp70-Ssa family as the sole source of Ssa protein display differences in global gene expression profiles. These changes in global gene expression may contribute significantly to the phenotypic differences observed between the Hsp70-Ssa family members.

**Electronic supplementary material:**

The online version of this article (doi:10.1186/1471-2164-15-194) contains supplementary material, which is available to authorized users.

## Background

A major class of heat shock proteins (Hsps) belong to the ubiquitous Hsp70 family, a diverse collection of 70 kDa chaperones that exist in various cellular compartments. Hsp70s perform essential housekeeping functions in protein folding, protein synthesis, translocation across membranes, protein degradation, assembly and disassembly of macromolecular complexes or aggregates, gene induction and apoptosis [1–4]. They are also involved in quality control process such as protein refolding after stress and control the activity of regulatory proteins in signal transduction pathways [5]. All of these cellular activities of Hsp70 depend upon its ability to interact with hydrophobic stretches of proteins in an ATP-dependent manner preventing non-productive interactions that would lead to aggregation and to promote protein refolding [6]. Hsp70 constitutes one of the most highly conserved and well-studied Hsps across species ranging from archaebacteria to plants and humans with the prokaryotic Hsp70 protein DnaK sharing approximately 50% amino acid similarity with eukaryotic Hsp70 proteins [7–10]. Various inter species expression studies of Hsp70 showed its conserved functional properties across distant species [11–15].

It had been suggested for a long time that the Hsp70 isoforms are functionally redundant and differ only by their spatio-temporal expression pattern. However, this was challenged by several findings in *Saccharomyces cerevisiae* (yeast) and higher eukaryotes demonstrating some functional specificity among Hsp70 isoforms [16, 17]. In yeast, the *hsp70* gene family comprises of fourteen genes, whose protein products share a sequence similarity of approximately 50-96%. Of these, nine are cytosolic and five are compartmental specific. The major cytosolic Hsp70 family in yeast is the Hsp70-Ssa (**S**tress **S**eventy sub-family **A**), which consists of four members of Ssa (Ssa1-4). These four isoforms are functionally redundant to some degree as expression of at least one family member is essential for growth [18]. Though other cytosolic Hsp70 sub-families cannot substitute for the survival function of Ssa sub-families, the four Ssa proteins can compensate for each other [19, 20]. Constitutively expressed Ssa1 and Ssa2 are 97% identical to each other and under optimal conditions Ssa2 is approximately fourfold more abundant than Ssa1 and depletion of Ssa2 induces expression of Ssa1, maintaining overall Hsp70 abundance. The heat-inducible Ssa3 and Ssa4 are 87% identical to each other and share an identity of 80% with Ssa1/2 [21]. The heat inducible isoforms are expressed under non-optimal growth conditions and help protect cells from the adverse effects of stress [22]. A major functional distinction between Ssa1 and Ssa2 exists in their effects on yeast prion propagation. The yeast [*PSI*^+^] and [*URE3*] prions are infectious forms of the Sup35 (involved in translation termination) and Ure2 (involved in regulating usage of poor nitrogen sources) proteins respectively [23]. Chaperones of various families, including Hsp70s, have been shown to play an integral part in modulating prion propagation [24, 25]. Overexpression of Ssa1 but not Ssa2, can cure [*URE3*] while depleting Ssa1 weakens [*PSI*^*+*^] but not [*URE3*] [26, 27]. The functional difference in Ssa1 or 2 in terms of prion propagation were found to be due to a single amino acid difference in the ATPase of these highly homologous proteins [28]. Hsp70 has also been implicated in biofilm production in yeast, which is another good example of functionally specificity among the Hsp70 isoforms [29]. Deletion of *ssa1* had a more adverse effect on biofilm formation in yeast compared to *ssa2* deletion. Additionally, Ssa3 and Ssa4 deletion enhanced the defects brought about by Ssa1/Ssa2 deletion suggesting cooperation between constitutive and inducible isoforms of Hsp70 [29]. Recently it was shown that Ssa1 (and probably other Ssa proteins) act as signal transducers mediating growth control through G1 cyclin abundance and activity, a process dependent on Ssa phosphorylation status at a highly conserved threonine residue in the ATPase domain [30]. Given the importance of the Hsp70 family in essential cellular functions and also that Hsp70 is a potential therapeutic target for a variety of human diseases, it is important to understand Hsp70s essential and non-essential roles within the cell and to characterize the functional difference between members of this chaperone family.

Here we make use of the genetically tractable yeast system. Using the plasmid shuffle technique we constructed yeast strains expressing either Ssa1, 2, 3 or 4 as the sole source of Hsp70-Ssa protein in the cell. To provide new insights into functional conservation and redundancy in the Hsp70-Ssa family, we carried out a comparative phenotypic analysis of these strains coupled with a global transcriptome analysis.

## Methods

### Strains, Plasmids and Genetic methods

The yeast strain used in this study was G402 (*MAT*a *ade2-1 SUQ5 kar1-1 his3 leu2 lys2 trp1 ura3 ssa1*::*KanMX*, *ssa2*::*HIS3*, *ssa3*::*TRP1*, *ssa4*::*URA3*-1f/pRDW10 [31]. All media used were as previously described by Loovers et al. [32]. Cultures were grown at 30°C unless indicated otherwise.

Plasmid pRDW10 contained in G402 strain [33] is a *URA3*-based, low-copy number centromeric plasmid with Ssa1, Ssa2, Ssa3 or Ssa4 as the sole source of Ssa in the cell under control of the *SSA2* promoter. The *SSA2* promoter was chosen as it is the only truly constitutive *SSA* promoter and allows comparative assessment of *SSA* gene and protein function without complicating factors such as heat shock induction. The *LEU2*-based plasmids pC210 (= pC210-Ssa1), pDCM62 (= pC210-Ssa2), pA3 (= pC210-Ssa3) and pA4 (= pC210-Ssa4) were described by Sharma *et al.* and Schwimmer and Masison [22, 26]. Plasmid pDCM90 is a *URA3*-based low-copy plasmid containing a gene for expression of a thermolabile bacterial luciferase LuxAB [34] on a *Cla*I-*Sma*I fragment. The pRS series of plasmids have been previously described by Sikorski et al. [35].

Monitoring of [*PSI*^*+*^] was carried out as described by Jones et al. [31]. Briefly, the presence of [*PSI*^*+*^] and the weak suppressor tRNA *SUQ5* in the strains were monitored as producing white colonies on media containing limiting amounts of adeneine, this is due to partial suppression and translation read through of the aberrant stop codon in the *ade2.1* allele [36, 37]. Nonsuppressed *ade2-1* mutants are adenine auxotrophs and are red when grown on adenine limiting media.

### Construction of G402 expressing only one Hsp70-Ssa family member

The plasmid shuffle technique was employed as essentially described by Loovers et al. [32] with minor modifications. The G402 strain contains the plasmid pRDW10, which contains a *URA3* marker and is the sole source of Ssa in the strain. G402 was transformed with a *LEU2* plasmid expressing either *SSA1*, 2, 3 or 4. Transformation plates were replica plated onto selective medium containing 5-fluoroorotic acid (5-FOA), a chemical that selects against *URA3* cells and hence against the presence of the pRDW10 plasmid. After 3 days at 30°C incubation, colonies were purified on medium lacking leucine and confirmed as uracil auxotrophs.

### Acquired thermotolerance Assay

Acquired thermotolerance assays were performed as described by Jung et al. [38] with minor modifications. Briefly, exponentially growing cultures were aliquoted and transferred to ice before exposing them to a temperature of 39°C for 1 hour to induce Hsp104. Subsequently, 1 ml volumes of cell aliquots were maintained in a 47°C shaking incubator and transferred to ice at the indicated time points. Serial dilution of the aliquots was carried out and the cells were spotted onto appropriate agar plates and incubated for 3 days at 30°C and growth were monitored over the period of time. The viability at time zero was set to 100%.

### Luciferase Assay

Luciferase refolding was assayed as essentially described by Parsell et al. [34]. Overnight cultures were diluted to an OD_600nm_ = 0.2 into the same selective medium and incubated at 37°C shaking for 30 minutes to induce expression of heat shock proteins. Subsquently, cellular luciferase activity of each strain was measured immediately using a FB 12 Luminometer (Berthold Detection Systems) following addition of 10 μl decanal, and the cells were transferred to 45°C with shaking for 1 hour. Cyclohexamide was added at a concentration of 10 μg/ml after 50 minutes at 45°C to prevent *de novo* synthesis of luciferase during the recovery period. Cultures were then further incubated at 45°C for 10 minutes. Cultures were shifted to 25°C for the recovery of cells from heat shock. Thereafter, luciferase refolding activity was measured as described above at 15 minute time intervals (for 1-hour) to check for luciferase recovery.

### Guanidine Curing

Routine curing of [*PSI*^+^] strains was done by streaking the yeast strains on YPD plates containing 3 mM Gdn-HCl and incubating the plates at 30°C for 3 days as essentially described by Jung et al. [33]. Cells were re-streaked onto YPD and red colonies were isolated. Both [*PSI*^+^] and [*psi*^-^] versions of the strains were maintained at 4°C and the stock was stored at -70°C.

### Western analysis

Western analysis was performed as described by Moran et al. [39]. Hsp70 monoclonal antibody was purchased from Cambridge Biosciences (Cambridge, UK) (SPA 822) and Hsp104 polyclonal antibody was a gift from John Glover (University of Toronto). Antibodies specific for Ssa1/2 and Ssa3/4 were a gift from Elizabeth Craig (University of Wisconsin, USA).

### RNA extraction

Total RNA was extracted from 5-ml cultures of the [*psi*^*-*^] yeast strains expressing either Ssa1, Ssa2, Ssa3 or Ssa4 grown overnight at 30°C. RNA was extracted using the Qiagen RNAesy kit as per manufacturer’s recommendation. For each strain, three replicates were used and each experiment was conducted twice. RNA was DNased-treated using TURBO DNA-free kit (Ambion, USA), according to the manufacturers recommendations. RNA concentrations were measured using a NanoDrop 1000 Spectrophotometer.

### Microarray analysis

For microarray analysis, equal amount of RNA from all three replicates from two separate experiments were pooled together and sent to Toray Industries, Japan. 3D-Gene^TM^ Yeast Oligo chip *S.cerevisiae* 6 k was used for the microarray analysis. The microarray analysis was done according to the manufacturer’s instructions. In brief, aRNA (amino allyl-labeled RNA) was synthesized from 1 μg total RNA with Amino Allyl MessageAmp^TM^ II aRNA Amplification kit (#1753: Ambion). 10 μg of aRNA was labelled with Cy5 Mono-ReactiveDyePack (PA25001:GE Health Care Bioscience) and 1 μg of labelled aRNA was hybridized at 37°C (250 rpm) for 16 hours. The washed and dried DNA chip was scanned by ScanArray Lite (Perkin Elmer). The obtained microarray images were quantified using GenePix Pro6.0 and the spot intensity was calculated by taking the median intensity of the foreground signals. The background signal intensity is derived by taking the mean signal intensity of the blank spots that excludes the top and bottom 5% signal intensities. The detected spots were defined as those that had signal intensity above the 95% upper confidence interval of the background signal intensity. For detected spots, their signal intensities were determined after subtracting with the mean background signal. For data comparison, the background-subtracted signal intensity was normalized using global normalization in which the median from each microarray was used. Global normalize values were LOG-transformed to linearize the data and a heat map was created using TIGR MultiExperiment Viewer (MeV) [40]. All microarray data from this study are complaint with Minimum Information About a Microarray Experiment (MIAME) and is publicly available through the NCBI's Gene Expression Omnibus (GEO) database (http://www.ncbi.nlm.nih.gov/geo) under the series record GSE32433.

### Real time RT-PCR analysis

Real-time RT-PCR analysis was used to verify the microarray results. Reverse transcription (RT) of total RNA was conducted as described by Ali et al. [41]. RT products were diluted to 200 μl and 2.5 μl were PCR-amplified in a 25 μl volume reaction containing 12.5 μl Premix Ex Taq^TM^ (Perfect Real Time) (Takara, Japan) and 100 nM each of forward and reverse transcript-specific primers (See Additional file 1: Table S1). PCR reactions were conducted in a Stratagene M×3000^TM^ real-time PCR machine (Stratagene, USA) and the programme consisted of 1 cycle of 95°C for 10 seconds, 40 cycles of 95°C for 5 seconds, 60°C for 30 seconds (annealing and polymerization) and 1 further cycle of 95°C for 60 seconds prior to melting curve analysis. Data were analysed using Stratagene M×3000^TM^ software (Stratagene, USA). All the 24 RT samples along with their minus-RT product amplified for the *S. cerevisiae ACT1* (YFL039C) gene and average threshold cycle (CT) for RT samples was 19.23 ± 0.17 while CT value for the minus-RT samples were ≤37.29. The expression patterns of the *ACT1* gene was used to normalise the RT-PCR data and the real-time quantification of target and housekeeping transcript accumulation was performed in separate reactions. The CT values obtained by real-time PCR (RT-qPCR) were used to calculate the accumulation of target gene (relative mRNA accumulation), relative to *ACT1* transcript, by 2^^-∆∆Ct^ method, where ∆∆Ct = (Ct, Target gene - Ct, *ACT1*) [42]. For each strain, three replicate samples were used and each experiment was conducted twice. The relative mRNA accumulation were LOG-transformed to linearize the data and a heat map of was created using TIGR MultiExperiment Viewer (MeV) [40].

### Statistical analysis

Normal distributon of data set was determined using the Ryan Joiner test [43] within Minitab (Minitab release 13.32^©^, 2000 Minitab Inc.). Non-normally distributed data were transformed to fit a normal distribution using the Johnson transformation [44] within Minitab (Minitab release 13.32^©^, 2000 Minitab Inc.). The homogeneity of datasets across replicate experiments was confirmed by one-tailed correlation analysis conducted using mean data values (non-normal data: Spearman Rank; normal data: Pearson product moment) conducted within the Statistical Package for the Social Sciences (SPSS 11.0, SPSS Inc.) (*r* ≥ 0.610; *P <* 0.050) [45]. Therefore, datasets from the replicate experiments were pooled for the purposes of further statistical analysis. The significance of treatment effects was analysed using SPSS by either (i) normally distributed data - one-way ANOVA with Post Hoc pair wise Least Significance Difference (LSD) comparisons (*P* = 0.050), or (ii) non-normally-distributed data – the Kruskal-Wallis H test. Correlations between mean values from different normally distributed datasets were calculated using Pearson product moment analysis.

## Results

### Confirmation of individual Hsp70-Ssa expression and prion propagation phenotype in strain G402

**Figure 1:**
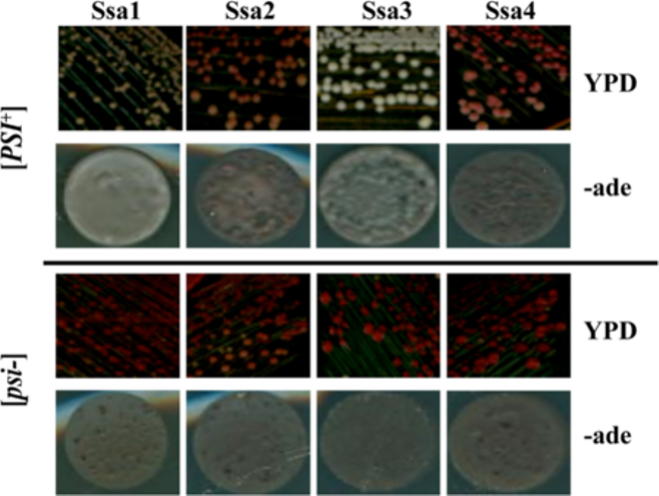
**Expression of individual members of the Ssa family in yeast and effects on [** ***PSI*** ^**+**^ **] phenotype.** Each member of the Ssa family was expressed as the sole source of Ssa in the yeast strain G402 by plasmid shuffle technique. Cells were streaked from 5-FOA onto YPD and following 3 days incubation at 30°C colonies were diluted in water and 20 μl spots were placed on -ade plates and incubated at 30°C for 3 days. Colour of the mutant strains ranged from white to pink, reflecting the varying degree of *ade 2–1* suppression due to the mutation. The extent of *ade 2–1* suppression is also reflected as density of growth on -ade plates. [psi-] variants were produced by curing [*PSI* ^+^] by streaking on 3 mM Gdn-HCl.

To assess the functions of individual members of the Ssa family, each member was expressed as a sole source of Ssa in the G402 strain using the plasmid shuffle technique. To confirm the identity of the strains constructed we verified Ssa chaperone protein expression and Hsp104 expression levels using Western blotting (Additional file 2: Figure S1) and prion propagation phenotype (Figure 1). As reported previously by Sharma et al. [21] the members of the Ssa family individually supported the growth of G402 (Figure 1). The [*PSI*^*+*^] strain was well maintained in cells expressing Ssa1 and particularly Ssa3 as the sole source of Ssa, as seen by the pigmentation on YPD and growth on adenine lacking media (Figure 1). The prion phenotype was also maintained in cells expressing Ssa2 but not to the same extent as Ssa1 or 3, while cells expressing only Ssa4 were seen to impair the propagation of [*PSI*^*+*^] the most dramatically (Figure 1), these findings are in agreement with Sharma and Masison [22]. Following plasmid shuffle all constructed strains were confirmed as [*PSI*^+^] by mating analysis and curing with Gdn-HCl. All Ssa family members were well expressed from the Ssa2 promoter and no major changes are seen in the expression levels of Hsp104, except perhaps for a minor increase when Ssa3 is the sole Ssa expressed (Additional file 2: Figure S1). There are no major changes in Ssa or Hsp104 expression levels in [*PSI*^+^] or [*psi*^-^] variants of strains used in this study (Additional file 3: Figure S2). Using well-established plate assays the curability of [*PSI*^+^] appeared similar for cells expressing different Hsp70-Ssas to the prion-curing agents Gdn-HCl, 6-aminophenanthridine and guanabenz acetate [46–48].

### Acquired thermotolerance activity of the Ssa family

**Figure 2:**
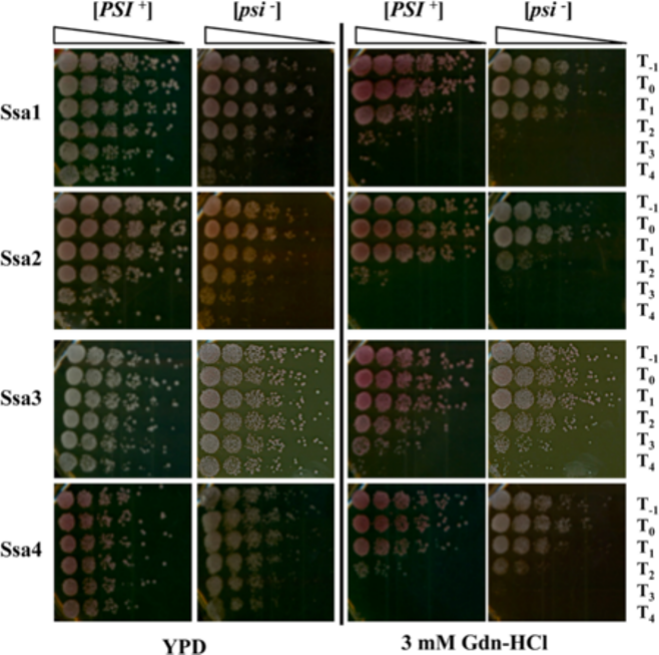
**Acquired thermotolerance assays for Ssa1-4.** Overnight culture was diluted in fresh YPD medium to an OD_600nm_ = 0.1 and then the cells were grown to exponential phase to a density of 3 × 10^6^ cells/ml. Cells were then re-suspended in fresh medium to a density of 5 × 10^6^ cells/ml. An aliquot (T_-1_) was then shifted to ice. The cultures were then incubated at 39°C for 1 hour to induce Hsp104 expression to protect against heat shock. Cells were then incubated at 47°C for 0, 10, 20, 30 and 40 minutes (T_0_-T_4_) and plated on YPD and 3 mM Gdn-HCl for comparative growth analysis. Representative spots shown in the figure are a neat concentration from a 1 in 5 serial dilution series. The plates were then incubated at 30°C for 3 days and were monitored for cellular thermotolerance. Cells without pre-treatment show virtually no growth at T_1_ (data not shown).

The survival rate of yeast post-lethal heat shock can be greatly improved by pre-treating cells with mild non-lethal heat shock, which stimulates elevation of heat shock proteins, including Hsp70 but primarily Hsp104 [8, 49]. Previously it had been demonstrated that yeast cells expressing only Ssa3 or Ssa4 (inducible Ssas) were more heat tolerant than those expressing only Ssa1 or Ssa2 (constitutive Ssas). It was suggested that this could be due to more efficient cooperation between inducible Hsp70s with Hsp104 compared to constitutive Hsp70s [21]. To address this question we carried out an acquired thermotolerance assay where the induced levels of Hsp104 expression provide resistance to prolonged exposure at high temperature. To assess the possible influence of the prion on survival, we assessed both [*PSI*^+^] and [*psi*^-^] variants. Using this assay we find that both Ssa3 and Ssa4 show enhanced acquired thermotolerance compared to Ssa1 and Ssa2, and this difference is much more pronounced in [*psi*^-^] variants (Figure 2, left panels). We assessed the contribution of Hsp104 to the enhanced thermotolerance of the inducible Hsp70s by plating surviving cells on medium containing 3 mM Gdn-HCl (Figure 2, right panels). Gdn-HCl inhibits *in vivo* activity of Hsp104 [38, 50]. Acquired thermotolerance was reduced for all strains compared to YPD medium, but the impact of Hsp104 inhibition was much less reduced for cells expressing Ssa3 as the sole Hsp70-Ssa. This suggests that Ssa3 has a more significant role in Hsp104-independent acquired thermotolerance compared to other Ssa proteins or perhaps there are some other differences for Ssa3 in terms of global transcriptomic or proteomic responses to heat shock or stress compared to other Ssas.

### *In vivo*protein refolding ability of individual Hsp70-Ssas

**Figure 3:**
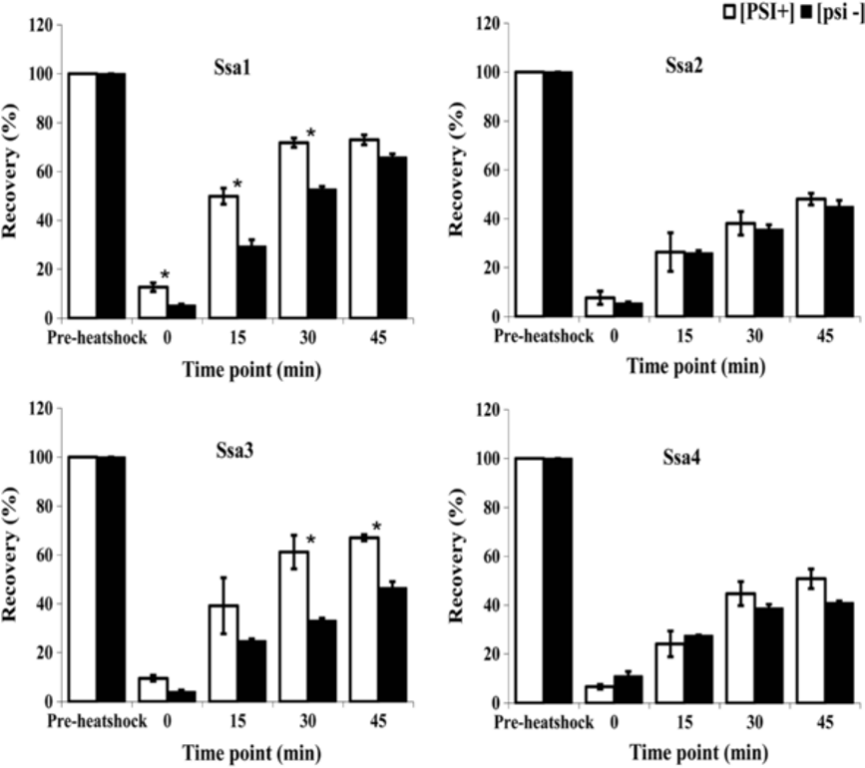
**Comparison of luciferase activity of [** ***PSI*** ^***+***^ **] and [** ***psi*** ^***-***^ **] versions of the Ssa family.** Overnight cultures were diluted in fresh SC medium lacking uracil to an OD_600nm_ = 0.1. The cultures were then shifted to 37°C for 30 minutes to induce the expression of Hsp104. After 30 minutes at 37°C, the cultures were shifted to 45°C for 1 hour. Cyclohexamide was added to the cultures after 50 minutes at 45°C to prevent any further synthesis of luciferase during the recovery period. Luciferase activity, expressed as a percentage of pre-heat shock activity, was measured at regular intervals during the recovery period of 45 minutes at 25°C. White [*PSI* ^+^], and black [*psi* ^-^]. (Bar indicates SEM. Value followed by * are significantly different at p ≤ 0.05).

We next assessed whether the *in vivo* protein refolding activity of the individual Ssas reflected the results from the acquired thermotolerance plate assays. We used the *in vivo* reactivation of thermo-labile bacterial luciferase as a measure of protein refolding ability of Hsp70-Ssa chaperones (Figure 3). In contrast to the thermotolerance plate assays, Ssa1 expressing cells were the most efficient at reactivating denatured luciferase with Ssa3 eventually reaching comparable levels with Ssa1 during the time course of the experiment. Ssa2 and Ssa4 showed similar activity but both reactivating approximately 20% less luciferase than Ssa1 and Ssa3 at the 45-minute time point. We also observed that the [*PSI*^+^] variants of Ssa1 and Ssa3 were more efficient at luciferase reactivation compared to [*psi*^-^] variants (Figure 3). The reason for this difference is unknown, but is unlikely to be due to alterations in chaperone levels (Additional file 2: Figure S1). As with the acquired thermotolerance plate assay, the presence of Gdn-HCl in cultures prior to carrying out the assay causes a major reduction in luciferase refolding activity (due to inhibition of Hsp104, data not shown).

### Differences in other stress phenotypes exhibited by the Ssa family

**Figure 4:**
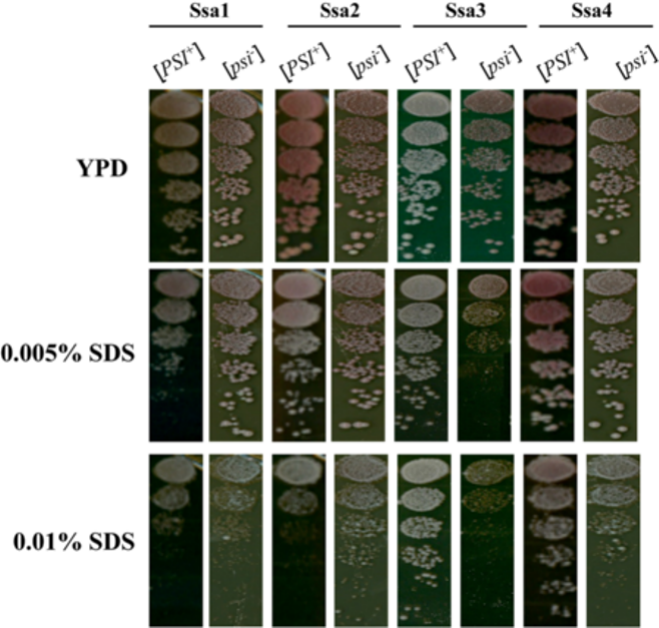
**Comparative growth analysis of the Ssa1-4 in response to SDS.** Overnight culture was diluted in fresh YPD medium to an OD_600nm_ = 0.1 and then the cells were grown to an exponential phase to a density of 3 × 10^6^ cells/ml. Cells were then re-suspended in fresh medium to a density of 5 × 10^6^ cells/ml and transferred to a microtitre plate. Representative spots shown in the figure are a neat concentration from a 1 in 5 serial dilution series. The plates were incubated for 3 days at 30°C.

**Figure 5:**
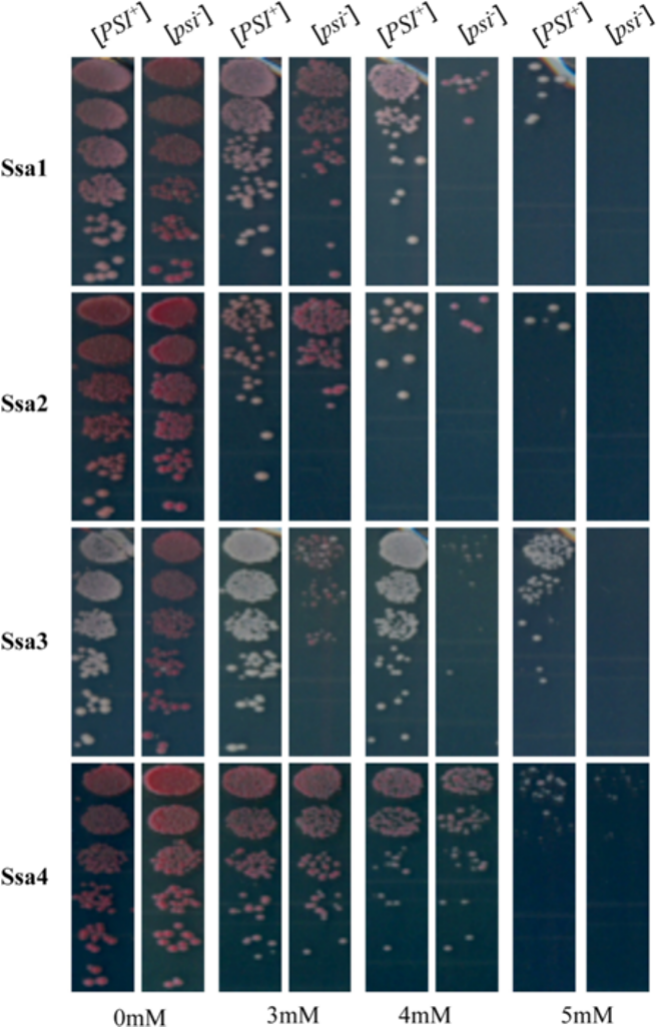
**Comparative growth analysis of Ssa1-4 in response to H**_**2**_ **O**_**2**_ **.** Overnight culture was diluted in fresh YPD medium to an OD_600nm_ = 0.1 and then the cells were grown to an exponential phase to a density of 3 × 10^6^ cells/ml. Cells were then re-suspended in fresh medium to a density of 5 × 10^6^ cells/ml and transferred to a microtitre plate. Representative spots shown in the figure are a neat concentration from a 1 in 5 serial dilution series. The plates were incubated for 3 days at 30°C.

In addition to differences in heat shock phenotypes of cells expressing different Ssa family members, we also observed differences in responses to the cell wall damaging agent, sodium dodecyl sulfate (SDS) (Figure 4) and the oxidative stress inducer, hydrogen peroxide (H_2_O_2_) (Figure 5).

Cells expressing Ssa4 exhibited greater resistance than other Ssas to SDS and the presence of [*PSI*^+^] also had an influence on SDS sensitivity for Ssa2 and Ssa3 expressing cells (Figure 4, bottom panels). Such an influence of [*PSI*^+^] has been observed previously for the closely related G600 strain [51], although in the case of G402 the reason for this effect is unknown.

Similarly, we also see that cells expressing ether Ssa3 and Ssa4 exhibit greater resistance to H_2_O_2_ compared to Ssa1 and Ssa2 (Figure 5). Additionally, a prion effect on sensitivity is also observed, however this effect is only seen for Ssa3 with the [*PSI*^+^] variant being more resistant than [*psi*^-^] at 3 mM H_2_O_2_ and higher. These results are reproducible but the reasoning behind this sensitivity profile is not known.

### Global transcription profile of yeast strains expressing individual Ssa’s

**Figure 6:**
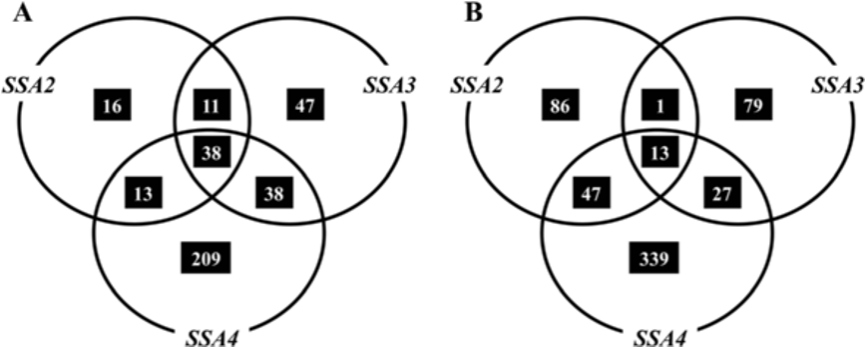
**Comparative transcriptome profiling of the Ssa family.** A Venn diagram representation of genes induced **(A)** or repressed **(B)** in different *∆ssa* strains. Analysis was performed relative to expression levels of G402 expressing *SSA1* as sole source of cytosolic Ssa protein.

Although the Hsp70-Ssa family carry out non-redundant cellular functions, increasing evidence suggests an array of Ssa family-specific functions exist. One such reason for the complex stress phenotypes exhibited in strains expressing individual Hsp70-Ssas could be due to changes in gene expression within the cell. We therefore assessed the global genome expression patterns for [*psi*^-^] variants for cells expressing individual Hsp70-Ssa family members. We used cells expressing Ssa1 as the control comparison. To assess global gene expression we used the highly sensitive Yeast 3D-Gene Microarray platform (Toray Industries, Japan). We identified a total of 78, 134 and 298 genes induced (>2-fold induction) and 147, 120 and 426 genes repressed (>2-fold repression) when Ssa2, Ssa3 and Ssa4 were expressed respectively as a sole source of Ssa in the cells compared to Ssa1 (Figure 6 and Additional file 4: Table S2). The strain with Ssa4 generated the highest differential expression with 209 genes induced and 339 genes repressed. In contrast, strains expressing only Ssa2 or Ssa3 proteins showed a relatively smaller number of induced (16 and 47 respectively) and repressed (86 and 79 respectively) genes.

An alternative way of assessing the strains expressing each individual Hsp70-Ssa family member is in terms of the gene deletions. Effectively, cells expressing Ssa1 can be viewed as a *∆ssa2∆ssa3∆ssa4* deletion strain, cells expressing Ssa2 as a *∆ssa1∆ssa3∆ssa4* deletion strain, cells expressing Ssa3 as a *∆ssa1∆ssa2∆ssa4* and cells expressing Ssa4 as a *∆ssa1∆ssa2∆ssa3* deletion strain. By comparing and identifying the co-regulated genes shared amongst the Ssa1/2/3/4 classes we can infer the regulation patterns for single and double deletions of each Ssa family member from our data. The overlapping circles in the Venn diagram (Figure 6) shows the numbers of shared genes between each of the Ssa family members and the gene identities are shown in Additional file 4: Table S2. Microarray data for these experiments is publicly available through the NCBI's GEO database with accession number GSE32433.

**Figure 7:**
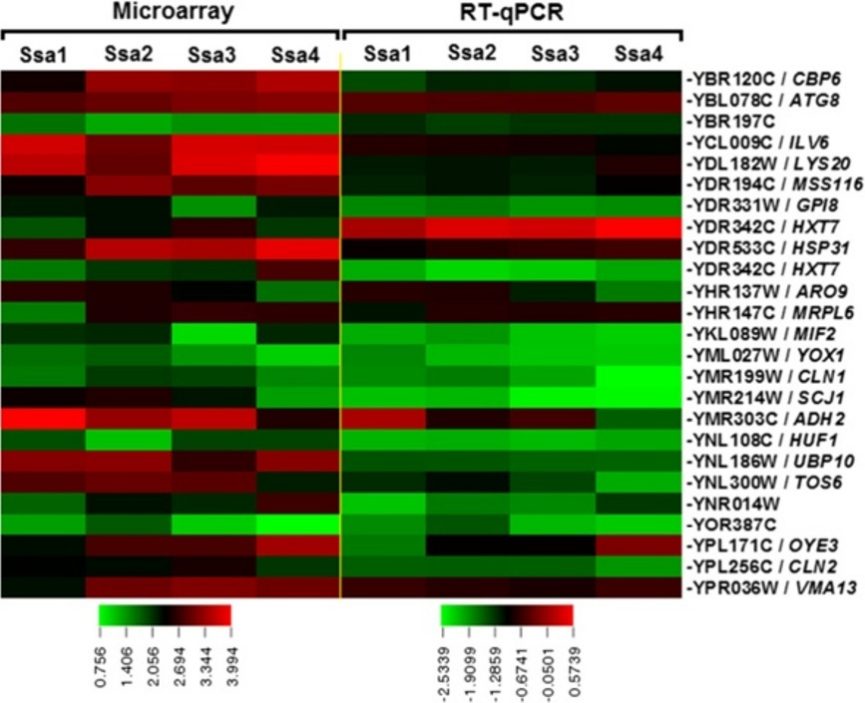
**Comparative overview of microarray and RT-qPCR analysis.** Twenty five genes were identified by microarray technique as being primed by yeast G402 strain carrying either Ssa1, 2, 3 or 4 as sole source of Ssa family protein. Both microarray and RT-qPCR analysis was conducted using total RNA extracted from 5-ml cultures of the [*psi* ^*-*^] yeast strains carrying Ssa1, 2, 3 or 4 grown overnight at 30°C. The 3D-Gene^TM^ Yeast Oligo chip *S.cerevisiae* 6 k used for the microarray analysis according to the manufacturer ’s instruction. Relative mRNA expression levels were quantified relative to that of the housekeeping gene *ACT1* (YFL039C) by 2^^-∆∆Ct^ method [43]. Bothe the global normalize value of microarray and the relative mRNA expression levels were LOG-transformed to linearize the data and the heat map of was created using TIGR MultiExperiment Viewer (MeV) [41]. Results are based on two experiments, each with three replicates.

RT-qPCR analysis was carried out to verify the microarray analysis. Twenty five genes were chosen based on their putative function and to represent a range of differential expression values among strains expressing Ssa1, Ssa2, Ssa3 or Ssa4 as the sole source of Ssa in the cells in a [*psi*^*-*^] background. The expression levels were normalised with a housekeeping gene *ACT1* and were LOG transformed. The comparative overview of the RT-qPCR and microarray results showed high degree of correlation and demonstrated the efficacy of both experiments (Figure 7 and Additional file 5: Table S3).

## Discussion

Expressing each member of the cytosolic Ssa family of Hsp70 as the sole source of Ssa to study the effect on prion propagation reinforces that yeast prion propagation and maintenance are influenced to varying degrees by different members. [*PSI*^*+*^] is the infectious prion form of the Sup35 protein (eRF3) and [*PSI*^*+*^] phenotype can be assessed by its ability to suppress the *ade2-1* premature *ochre* mutation present within *ADE2* gene in strain G402. Sup35 protein (eRF3) under normal conditions in the cell encodes a translation release factor [52, 53]. Aggregation of the Sup35 in [*PSI*^*+*^] cells causes nonsense suppression because of the depletion of Sup35 protein into an insoluble prion aggregate that is no longer functional. Thus, the strength of prion phenotype is directly proportional to the amount of Sup35 protein that has been depleted to insoluble prion aggregates [33]. Ssa family members differed in their abilities to propagate [*PSI*^*+*^], with Ssa3 being the most effective member in maintenance and propagation of [*PSI*^*+*^], compared to other Ssa proteins. This result is consistent with a previous study by Sharma et al. [21]. Earlier studies by Schwimmer *et al.* and Roberts *et al.*[26, 27] also found different effects of the Ssa family on [*URE3*] propagation. For example, overexpression of Ssa1 but not Ssa2 can cure [*URE3*], whereas a mutation in Ssa2 but not Ssa1 impairs [*URE3*] propagation. Recently, corresponding changes of a single ATPase domain residue (A83G in Ssa1, G83A in Ssa2) in Ssa1 or Ssa2 was able to allow these Hsp70-Ssa family members to acquire the prion propagation behaviour of the other [28]. It appears that the evolution and occurrence of multiple Hsp70s within a species may be due to modified regulation of Hsp70 substrate-binding activity rather than changes to range of substrate that is recognised [28]. In *S. cerevisiae*, the activity of Ssa proteins is regulated by J-proteins [54] and NEFs such as Fes1 and Sse1/2 [55–60]. It has not been systematically tested whether the different Ssa proteins have different affinities for J-proteins and NEFs but remains an attractive possibility that this could partly explain different prion phenotypes when members of the Ssa family are individually expressed as a sole source of Ssa in the cell.

The Hsp104 protein influences cell survival under prolonged exposure to high temperatures [24]. At elevated temperatures, Hsp104 activity allows cells to survive by resolubilising heat-denatured proteins [34]. Apart from Hsp104, refolding of misfolded protein requires the activity of additional chaperone proteins such as Hsp70 and Hsp40. Thus Hsp104, in conjunction with Hsp70 and Hsp40, constitutes a protein disaggregation machinery leading to the resolubilisation of protein aggregates [61]. Both the constitutive and heat-inducible isoforms of Hsp70 functioned well with Hsp104 in refolding thermally denatured luciferase but preferentially, Ssa1 and Ssa3 were more active *in vivo* for luciferase refolding than Ssa2 and Ssa4 (Figure 3). Such differences may be reflected by the specific Hsp70 component of the protein disaggregation machinery. It has been proposed that heat inducible Hsp70s may function more efficiently with Hsp104 than constitutive Hsp70s [21]. However, this does not appear to be the case. Ssa3 clearly provides better levels of acquired thermotolerance than other Ssas (Figure 2), but even when cells are allowed to recover on medium that inhibits Hsp104 activity (GdnHCl plates), cells expressing Ssa3 still recover remarkably well. This result suggests that for cells expressing Ssa3 as the sole Hsp70-Ssa, there is a greater impact of factors other than Hsp104 in determining levels of acquired thermotolerance. Additionally, the assessment of refolding activity of a model substrate such as luciferase does not necessarily correlate well with the *in vivo* refolding activity required for survival of heat stress.

Further complicating the interpretation of the luciferase refolding and acquired thermotolerance plate assays is the fact that Ssa1 is the most efficient in refolding denatured luciferase but does match or improve upon Ssa3 in the plate assay. Additionally, the presence of [*PSI*^+^] caused a clear and reproducible increase in efficiency in luciferase refolding in the Ssa1 and Ssa3 expressing strains (Figure 3), which did not translate to increased survival in the acquired thermotolerance plate assays (Figure 2). Taken together these results also suggest a significant influence on levels of acquired thermotolerance from other cellular sources in addition to Hsp104.

Following further phenotypic stress analysis we also identified differences between the Ssas in response to oxidative and cell wall stress inducing agents (Figures 4 and 5). Of particular note is the trend for the inducible Ssas to provide better protection against both H_2_O_2_ and SDS. However, again a complex stress response is observed, as the presence of [*PSI*^+^] appears to influence sensitivity to SDS and to H_2_O_2_, particularly when only Ssa3 is expressed. The reason for this prion influence is not known, but has been observed before in the closely related strain G600 [51]. Given that cells expressing only Ssa3 have a much stronger prion phenotype (Figure 1b) then prion-specific effects may be expected in these cells compared to others expressing different Ssa family members.

The different and complex prion and stress phenotypes exhibited by cells expressing individual members of the Hsp70-Ssa family led us to hypothesize that in addition to any inherent functional differences that may exist amongst these highly homologous proteins, the potential existed for indirect phenotypic influences caused by changes in global gene expression. This hypothesis appears to hold true as under non-stress conditions and when expressed from the same promoter, there are many differences in the expression levels of a variety of genes between cells expressing different Hsp70-Ssa family members (Figure 6 and Additional file 4: Table S2). The changes in expression levels are in genes that are involved in a diverse set of cellular functions that would be predicted to influence a variety of cellular phenotypes (Additional file 4: Table S2). The full cellular implications for these changes in gene expression are difficult to interpret, but some inferences can be made in respect of the stress phenotypes we have observed in this study. For instance, cells expressing either Ssa2 or Ssa4 showed a 3-fold repression of *CTA1. CTA1* encodes catalase A, which is involved in hydrogen peroxide detoxification in the peroxisomal and mitochondrial matrices [62, 63]. Additionally, *CTT1*, a catalase that has a role in protection of cell from oxidative damage caused by hydrogen peroxide [64], was over 2-fold repressed in Ssa2. Repression of these genes could be an influencing factor in the comparative sensitivity to H_2_O_2_ of cells expressing solely Ssa2. Also, the transcriptional profiling of the Ssa family also revealed that the *TRX2* gene, which confers resistance to H_2_O_2_[65], was among the top listed induced genes in Ssa4 strain and this may provide an explanation for the comparative resistance to H_2_O_2_ displayed by cells expressing solely Ssa4. In addition to this, two other genes, *GPX2*, which is a glutathione peroxidise that is induced by oxidative stress [66] and *GRX1*, which is a glutaredoxin were induced (3.13 and 2.49 fold respectively) only in Ssa4 expressing cells. Glutaredoxins are the source primary defenses against mixed disulfides formed following oxidative damage to proteins [67].

Further linking phenotype to possible changes in gene expression, we observed changes in genes involved in cell wall integrity (CWI) signalling in Ssa3 expressing cells. The sensitivity of the [*psi*^-^] variant of Ssa3 to 0.005% SDS (cell wall damaging agent) compared to [*psi*^-^] variant of other Ssas, suggests a possible reduced efficiency in cell wall integrity signalling in the Ssa3 strain. The sensitivity displayed by cells expressing Ssa3 may be explained by the suppression of two genes, *LRG1* and *PMT6. LRG1* is a GTPase - activating protein (GAP) that is involved in the Pkc1-mediated signalling pathway that controls cell wall integrity [68], while *PMT6* belongs to a family of protein mannosyltransferases that catalyses the transfer of mannose from dolichyl phosphate-D-mannose to protein serine/threonine residues of secretory proteins, a reaction essential for cell wall rigidity and cell integrity [69]. Both of these genes were repressed in only Ssa3 expressing cells by over 2-fold (Additional file 4: Table S2). Our findings implicate a possible role for Hsp70-Ssa family in the CWI signalling pathway.

A previous study comparing the global gene expression changes in yeast upon mild heat shock to those for yeast cells deleted for both *SSA1* and *SSA2*, identified differential expression of groups of genes that are very distinct from those identified in our analysis [70]. Significant differences in strain background and *SSA* deletion status exists between these studies and therefore a direct comparison between global gene expression data is not possible.

## Conclusion

While it is clear that Hsp70-Ssa family members provide redundant functions within the yeast cell, it is also evident that within groups of highly homologous Hsp70s, family members may evolve to be much more efficient at aspects of these shared functions or have even developed new specific roles within the cell. However, a major conclusion from this study is that while some phenotypic differences observed between cells expressing different Hsp70-Ssa family members may result in part from intrinsic functional differences between Hsp70s, a significant contribution to strain phenotype may also be attributed to major changes in global gene expression within the cell.

### Availability of Supporting Data

Microarray data is publicly available through the NCBI's Gene Expression Omnibus (GEO) database http://www.ncbi.nlm.nih.gov/geo/query/acc.cgi?acc=GSE32433.

## Electronic supplementary material

Additional file 1: Table S1: Primers used for RT-qPCR. (PDF 73 KB)

Additional file 2: Figure S1: Expression of individual Hsp70-Ssa family members in yeast. To confirm expression of individual Ssa family members we used Western blotting with antibodies recognising Hsp70-Ssa (top panel), Ssa1 or Ssa2 only and Ssa3 or Ssa4 only. We also assessed Hsp104 expression in these cells. Ssa1/2 and Ssa3/4 specific antibodies were a gift from Elizabeth Craig. Hsp104 antibody was a gift from John Glover. Loading control is membrane stained with amido black. (PDF 229 KB)

Additional file 3: Figure S2: Relative abundance of Hsp70 and Hsp104 in yeast cells expressing. individual Ssa’s. Western blot analysis was performed to examine the abundance of Hsp70 and Hsp104. Blots probed with anti-Hsp70 antibodies (SPA822, Cambridge Biosciences) were stripped and re-probed with anti-Hsp104 antibodies (gift from John Glover). Membrane was then stained by amido black as a loading and transfer control, are shown (Load). The Ssa strains are indicated on the top. (PDF 237 KB)

Additional file 4: Table S2: Systematic and standard names and descriptions are based on Saccharomyces Genome Database (SGD) http://www.yeastgenome.org; **Orange cells show the data more than 100. (XLS 350 KB)

Additional file 5: Table S3: Comparative overview of fold change for microarray and qPCR of 25 genes. (PDF 61 KB)

## References

[1] Nollen EAA, Morimoto RI (2002). Chaperoning signaling pathways: molecular chaperones as stress-sensing heat shock proteins. J Cell Sci.

[2] Sangster TA, Lindquist S, Queitsch C (2004). Under cover: causes, effects and implications of Hsp90 - mediated genetic capacitance. Bioessays.

[3] De Los RP, Ben-Zvi A, Slutsky O, Azem A, Goloubinoff P (2006). Hsp70 chaperones accelerate protein translocation and the unfolding of stable protein aggregates by entropic pulling. Proc Natl Acad Sci USA.

[4] Floer M, Bryant GO, Ptashne M (2008). Hsp90/70 chaperones are required for rapid nucleosome removal upon induction of the *GAL* genes of yeast. Proc Natl Acad Sci USA.

[5] Mayer M, Bukau B (2005). Hsp70 chaperones: cellular functions and molecular mechanism. Cell Mol Life Sci.

[6] Sharma D, Masison DC (2009). Hsp70 structure, function, regulation and influence on yeast prions. Protein Pept. Lett.

[7] Daugaard M, Rohde M, Jäättelä M (2007). The heat shock protein 70 family: Highly homologous proteins with overlapping and distinct functions. FEBS letters.

[8] Lindquist S, Craig E (1988). The heat-shock proteins. Annu Rev Genet.

[9] Gupta RS, Singh B (1994). Phylogenetic analysis of 70 kDa heat shock protein sequences suggests a chimeric origin for the eukaryotic cell nucleus. Curr Biol.

[10] Hunt C, Morimoto RI (1985). Conserved features of eukaryotic *hsp70* genes revealed by comparison with the nucleotide sequence of human *hsp70*. Proc Natl Acad Sci USA.

[11] Pelham H (1984). Hsp70 accelerates the recovery of nucleolar morphology after heat shock. EMBO J.

[12] Li GC, Li L, Liu YK, Mak JY, Chen L, Lee W (1991). Thermal response of rat fibroblasts stably transfected with the human 70-kDa heat shock protein-encoding gene. Proc Natl Acad Sci USA.

[13] Li G, Li L, Liu R, Rehman M, Lee W (1992). Protection from thermal stress by human hsp70 with or without its ATP-binding domain. Proc Natl Acad Sci USA.

[14] Jäättelä M, Wissing D, Bauer PA, Li GC (1992). Major heat shock protein hsp70 protects tumor cells from tumor necrosis factor cytotoxicity. EMBO J.

[15] Tutar Y, Song Y, Masison DC (2006). Primate chaperones Hsc70 (constitutive) and Hsp70 (induced) differ functionally in supporting growth and prion propagation in *Saccharomyces cerevisiae*. Genetics.

[16] Gao B, Biosca J, Craig EA, Greene LE, Eisenberg E (1991). Uncoating of coated vesicles by yeast hsp70 proteins. J Biol Chem.

[17] Kabani M, Martineau CN (2008). Multiple hsp70 isoforms in the eukaryotic cytosol: mere redundancy or functional specificity?. Curr Genomics.

[18] Werner-Washburne M, Stone DE, Craig EA (1987). Complex interactions among members of an essential subfamily of hsp70 genes in *Saccharomyces cerevisiae*. Mol Cell Biol.

[19] Boorstein WR, Ziegelhoffer T, Craig EA (1994). Molecular evolution of the Hsp70 multigene family. J Mol Evol.

[20] James P, Pfund C, Craig EA (1997). Functional specificity among Hsp70 molecular chaperones. Science.

[21] Sharma D, Martineau CN, Le Dall MT, Reidy M, Masison DC, Kabani M (2009). Function of *SSA* subfamily of Hsp70 within and across species varies widely in complementing *Saccharomyces cerevisiae* cell growth and prion propagation. PloS one.

[22] Sharma D, Masison DC (2008). Functionally redundant isoforms of a yeast Hsp70 chaperone subfamily have different antiprion effects. Genetics.

[23] Perrett S, Jones GW (2008). Insights into the mechanism of prion propagation. Curr Opin Struct Biol.

[24] Jones GW, Tuite MF (2005). Chaperoning prions: the cellular machinery for propagating an infectious protein?. Bioessays.

[25] Reidy M, Masison DC (2011). Modulation and elimination of yeast prions by protein chaperones and co-chaperones. Prion.

[26] Schwimmer C, Masison DC (2002). Antagonistic interactions between yeast [*PSI*^*+*^] and [*URE3*] prions and curing of [*URE3*] by Hsp70 protein chaperone Ssa1p but not by Ssa2p. Mol Cell Biol.

[27] Tibor Roberts B, Moriyama H, Wickner RB (2004). [*URE3*] prion propagation is abolished by a mutation of the primary cytosolic Hsp70 of budding yeast. Yeast.

[28] Sharma D, Masison DC (2011). Single methyl group determines prion propagation and protein degradation activities of yeast heat shock protein Hsp70 chaperones Ssa1p and Ssa2p. Proc Natl Acad Sci USA.

[29] Martineau CN, Beckerich JM, Kabani M (2007). Flo11p-independent control of “mat” formation by hsp70 molecular chaperones and nucleotide exchange factors in yeast. Genetics.

[30] Truman AW, Kristjansdottir K, Wolfgeher D, Hasin N, Polier S, Zhang H, Perrett S, Prodromou C, Jones GW, Kron SJ (2012). CDK-dependent Hsp70 Phosphorylation controls G1 cyclin abundance and cell-cycle progression. Cell.

[31] Jones GW, Masison DC (2003). *Saccharomyces cerevisiae* Hsp70 mutations affect [*PSI*^*+*^] prion propagation and cell growth differently and implicate Hsp40 and tetratricopeptide repeat cochaperones in impairment of [*PSI*^*+*^]. Genetics.

[32] Loovers HM, Guinan E, Jones GW (2007). Importance of the Hsp70 ATPase domain in yeast prion propagation. Genetics.

[33] Jung G, Jones G, Wegrzyn RD, Masison DC (2000). A Role for Cytosolic Hsp70 in Yeast [*PSI*^*+*^] Prion Propagation and [*PSI*^*+*^] as a Cellular Stress. Genetics.

[34] Parsell DA, Kowal AS, Singer MA, Lindquist S (1994). Protein disaggregation mediated by heat-shock protein Hspl04. Nature.

[35] Sikorski RS, Hieter P (1989). A system of shuttle vectors and yeast host strains designed for efficient manipulation of DNA in *Saccharomyces cerevisiae*. Genetics.

[36] Liebman SW, Stewart JW, Sherman F (1975). Serine substitutions caused by an ochre suppressor in yeast. J Mol Biol.

[37] Cox BS (1965). "ψ" a cytoplasmic suppressor of super-suppressor in yeast. Heredity.

[38] Jung G, Masison DC (2001). Guanidine hydrochloride inhibits Hsp104 activity in vivo: a possible explanation for its effect in curing yeast prions. Curr Microbiol.

[39] Moran C, Kinsella GK, Zhang ZR, Perrett S, Jones GW (2013). Mutational Analysis of Sse1 (Hsp110) Suggests an Integral Role for this Chaperone in Yeast prion Propagation *In Vivo*. G3.

[40] Saeed A, Sharov V, White J, Li J, Liang W, Bhagabati N, Braisted J, Klapa M, Currier T, Thiagarajan M (2003). TM4: a free, open-source system for microarray data management and analysis. Biotechniques.

[41] Ali SS, Nugent B, Mullins E, Doohan FM (2013). Insights from the Fungus *Fusarium oxysporum* Point to High Affinity Glucose Transporters as Targets for Enhancing Ethanol Production from Lignocellulose. PloS one.

[42] Livak KJ, Schmittgen TD (2001). Analysis of Relative Gene Expression Data Using Real-Time Quantitative PCR and the 2 ^-ΔΔCT^ Method. Methods.

[43] Ryan T, Joiner B (1976). Normal probability plots and tests for normality. Tech. Rep.

[44] Johnson NJ (1978). Modified t tests and confidence intervals for asymmetrical populations. J Am Stat Soc.

[45] Snedecor G, Cochran W (1980). Statistical Methods.

[46] Tuite M, Mundy C, Cox B (1981). Agents that cause a high frequency of genetic change from [*PSI*^*+*^] to [*psi*^-^] in *Saccharomyces cerevisiae*. Genetics.

[47] Bach S, Talarek N, Andrieu T, Vierfond J-M, Mettey Y, Galons H, Dormont D, Meijer L, Cullin C, Blondel M (2003). Isolation of drugs active against mammalian prions using a yeast-based screening assay. Nat Biotechnol.

[48] Tribouillard-Tanvier D, Béringue V, Desban N, Gug F, Bach S, Voisset C, Galons H, Laude H, Vilette D, Blondel M (2008). Antihypertensive drug guanabenz is active in vivo against both yeast and mammalian prions. PloS one.

[49] Landry J, Bernier D, Chrétien P, Nicole LM, Tanguay RM, Marceau N (1982). Synthesis and degradation of heat shock proteins during development and decay of thermotolerance. Cancer Res.

[50] Ferreira PC, Ness F, Edwards SR, Cox BS, Tuite MF (2001). The elimination of the yeast [*PSI*^*+*^] prion by guanidine hydrochloride is the result of Hsp104 inactivation. Mol Microbiol.

[51] Fitzpatrick DA, O'Brien J, Moran C, Hasin N, Kenny E, Cormican P, Gates A, Morris DW, Jones GW (2011). Assessment of Inactivating Stop Codon Mutations in Forty *Saccharomyces cerevisiae* Strains: Implications for [*PSI*^*+*^] Prion-Mediated Phenotypes. PloS one.

[52] Stansfield I, Jones K, Kushnirov V, Dagkesamanskaya A, Poznyakovski A, Paushkin S, Nierras C, Cox B, Ter-Avanesyan M, Tuite M (1995). The products of the *SUP45* (eRF1) and *SUP35* genes interact to mediate translation termination in *Saccharomyces cerevisiae*. EMBO J.

[53] Zhouravleva G, Frolova L, Le Goff X, Le Guellec R, Inge-Vechtomov S, Kisselev L, Philippe M (1995). Termination of translation in eukaryotes is governed by two interacting polypeptide chain release factors, eRF1 and eRF3. EMBO J.

[54] Sahi C, Craig EA (2007). Network of general and specialty J protein chaperones of the yeast cytosol. Proc Natl Acad Sci USA.

[55] Shaner L, Wegele H, Buchner J, Morano KA (2005). The yeast Hsp110 Sse1 functionally interacts with the Hsp70 chaperones Ssa and Ssb. J Biol Chem.

[56] Yam AYW, Albanèse V, Lin HTJ, Frydman J (2005). Hsp110 cooperates with different cytosolic Hsp70 systems in a pathway for *de novo* folding. J Biol Chem.

[57] Dragovic Z, Broadley SA, Shomura Y, Bracher A, Hartl FU (2006). Molecular chaperones of the Hsp110 family act as nucleotide exchange factors of Hsp70s. EMBO J.

[58] Raviol H, Sadlish H, Rodriguez F, Mayer MP, Bukau B (2006). Chaperone network in the yeast cytosol: Hsp110 is revealed as an Hsp70 nucleotide exchange factor. EMBO J.

[59] Kabani M, Beckerich JM, Brodsky JL (2002). Nucleotide exchange factor for the yeast Hsp70 molecular chaperone Ssa1p. Mol Cell Biol.

[60] Dragovic Z, Shomura Y, Tzvetkov N, Hartl FU, Bracher A (2006). Fes1p acts as a nucleotide exchange factor for the ribosome-associated molecular chaperone Ssb1p. Biol Chem.

[61] Glover JR, Lindquist S (1998). Hsp104, Hsp70, and Hsp40: A Novel Chaperone System that Rescues Previously Aggregated Proteins. Cell.

[62] Cohen G, Fessl F, Traczyk A, Rytka J, Ruis H (1985). Isolation of the catalase A gene of *Saccharomyces cerevisiae* by complementation of the cta1 mutation. MGG.

[63] Petrova V, Drescher D, Kujumdzieva A, Schmitt M (2004). Dual targeting of yeast catalase A to peroxisomes and mitochondria. Biochem J.

[64] Grant CM, Perrone G, Dawes IW (1998). Glutathione and Catalase Provide Overlapping Defenses for Protection against Hydrogen Peroxide in the Yeast *Saccharomyces cerevisiae*. Biochem Biophys Res Commun.

[65] Morano KA, Grant CM, Moye-Rowley WS (2012). The response to heat shock and oxidative stress in *Saccharomyces cerevisiae*. Genetics.

[66] Inoue Y, Matsuda T, Sugiyama K-i, Izawa S, Kimura A (1999). Genetic analysis of glutathione peroxidase in oxidative stress response of *Saccharomyces cerevisiae*. J Biol Chem.

[67] Luikenhuis S, Perrone G, Dawes IW, Grant CM (1998). The yeast *Saccharomyces cerevisiae* contains two glutaredoxin genes that are required for protection against reactive oxygen species. Mol Biol Cell.

[68] Lorberg A, Schmitz H-P, Jacoby J, Heinisch J (2001). Lrg1p functions as a putative GTPase-activating protein in the Pkc1p-mediated cell integrity pathway in *Saccharomyces cerevisiae*. Mol. Genet. Genomics.

[69] Gentzsch M, Tanner W (1996). The PMT gene family: protein O-glycosylation in *Saccharomyces cerevisiae* is vital. EMBO J.

[70] Matsumoto R, Akama K, Rakwal R, Iwahashi H (2005). The stress response against denatured proteins in the deletion of cytosolic chaperones SSA1/2 is different from heat-shock response in *Saccharomyces cerevisiae*. BMC Genomics.

