# Supplementary material for: Global transcript and phenotypic analysis of yeast cells expressing Ssa1, Ssa2, Ssa3 or Ssa4 as sole source of cytosolic Hsp70-Ssa chaperone activity
Source: BMC Genomics. 2014 Mar 14;15(1):194. doi: 10.1186/1471-2164-15-194 (PMC4022180; doi:10.1186/1471-2164-15-194)
Supplement: Supplementary file 1 — Additional file 1: Table S1: Primers used for RT-qPCR. (PDF 73 KB) [file 12864_2013_7032_MOESM1_ESM.pdf]

Table S1: Primers used for RT-qPCR

Systematic gene name[a]	Primers	Oligonucleotide
YFL039C	ACT1-F	GGGTTTGGAATCTGCC
	ACT1-R	CCACCAATCCAGACGGAG
YBR120C	CBP6-F	TAATTAACAGAACTTCCGGTCCA
	CBP6-R	TACCGTCAACGGCATTTATTAAC
YBR197C	YBR197C-F	TTCCACCAACAGAAGGAAATCTA
	YBR197C-R	GAGTGCATTGTTATACGGCTACC
YNL300W	TOS6-F	CTCTACTCTCTCCACCGTTGCT
	TOS6-R	GTGGTGTTAGCTTGGCTACTGAT
YML027W	YOX1-F	ATCACCAAGCTTCACTAATCCAA
	YOX1-R	TCGTGTGATTCATCAACTGTTTC
YMR199W	CLN1-F	CTAATCAAGATCAAGGCCCTTTT
	CLN1-R	CTATTGTAGAGGCCAGTTGCAGT
YPL256C	CLN2-F	TATGTGGTAGCACCGTTAGTGTG
	CLN2-R	TCCATGGTTAATGAAGCTTGTCT
YHR147C	MRPL6-F	TAGGTGTTACTGAGGGGCATCT
	MRPL6-R	CTCTGGTGGATGGAATTTTCTC
YBL078C	ATG8-F	CCGTAGGGCAATTTGTTTATGT
	ATG8-R	ACAAAAACCCGTCCTTATCCTT
YDR533C	HSP31-F	CAAGACCGGTGTTTTTGTTGTA
	HSP31-R	AATTTGGTAATCATCGGCATTC
YPR036W	VMA13-F	CCGTGATACCAGAGTATCGTGA
	VMA13-R	AAACTGGGTTAAAGGTCAGCAA
YDR194C	MSS116-F	ATTAGGTTTGAGCCGTAGTCCA
	MSS116-R	ACGGTTCGTCATCGTAATCTCT
YPL171C	OYE3-F	TTTAGCCATCCATGATTGTCAG
	OYE3-R	GATAGAATTCTTAGCCGCATGG
YHR094C	HXT1-F	CCAGCCATGTTTCAACGTACTA
	HXT1-R	GGTCAACGGTGTACAGAGAACA
YNR014W	YNR014W-F	TCACTACTGCCCTCTTCTAGGC
	YNR014W-R	GTACGAGACGGACCTTTTCATC

Table S1 continued…….

Systematic gene name	Primers	Oligonucleotide
YNL108C	HUF1-F	ACCGGTATTGATAACGATGTCC
	HUF1-R	CTCCTACACCACGATCGACATA
YCL009C	ILV6-F	AACACCGAGGTCAAAGACCTAA
	ILV6-R	CTGGCCATCACTAGCTCTCTTT
YKL089W	MIF2-F	GTAGAAGAGGAGGCTGAGGTGA
	MIF2-R	ATGTCAAGAACGGGTTTATTGG
YDR331W	GPI8-F	AGGCTGTTAACGGATAGATGGA
	GPI8-R	TTTTCGTACATTTGCTGGAATG
YNL186W	UBP10-F	AAACTTGGGTTCTCCATTAGCA
	UBP10-R	CAGAAATTTTGGAGCTGGAATC
YMR214W	SCJ1-F	TGATCAAGGTCCAGGAAAAACT
	SCJ1-R	ACCACACATCTGTTGAATCTGC
YHR137W	ARO9-F	ACAGAGAGTCCCAGGGTATTGA
	ARO9-R	TCAACAATGAGGAAATCGTGTC
YOR387C	YOR387C-F	ATTGGTGATGCTATGGTTCCTT
	YOR387C-R	GGGGTGCTCAAAATAACAGAAG
YMR303C	ADH2-F	CATTGTTAGCGCAGTCGTTAAG
	ADH2-R	ACAACGTGGTTGAAGACATCAG
YDL182W	LYS20-F	ACTGCTGCTAAACCAAATCCAT
	LYS20-R	ATGTAGTCCACACCGAAATCGT
YDR342C	HXT7-F	GGGCTGTTTGGTCTTCATGT
	HXT7-R	ACCTCTTCTGGATGGTGGAA

Primers were design using the Primer3 software (version 0.4.0; http://frodo.wi.mit.edu/primer3/). Primer specificity was confirmed by melting curve analyses of real-time RT-PCR products (81 cycles of 55°C for 30s).

[a]Systematic names and the sequence was used for design of primers were based on *Saccharomyces* Genome Database (http://www.yeastgenome.org/).
