# Supplementary material for: Global transcript and phenotypic analysis of yeast cells expressing Ssa1, Ssa2, Ssa3 or Ssa4 as sole source of cytosolic Hsp70-Ssa chaperone activity
Source: BMC Genomics. 2014 Mar 14;15(1):194. doi: 10.1186/1471-2164-15-194 (PMC4022180; doi:10.1186/1471-2164-15-194)
Supplement: Supplementary file 2 — Additional file 2: Figure S1: Expression of individual Hsp70-Ssa family members in yeast. To confirm expression of individual Ssa family members we used Western blotting with antibodies recognising Hsp70-Ssa (top panel), Ssa1 or Ssa2 only and Ssa3 or Ssa4 only. We also assessed Hsp104 expression in these cells. Ssa1/2 and Ssa3/4 specific antibodies were a gift from Elizabeth Craig. Hsp104 antibody was a gift from John Glover. Loading control is membrane stained with amido black. (PDF 229 KB) [file 12864_2013_7032_MOESM2_ESM.pdf]

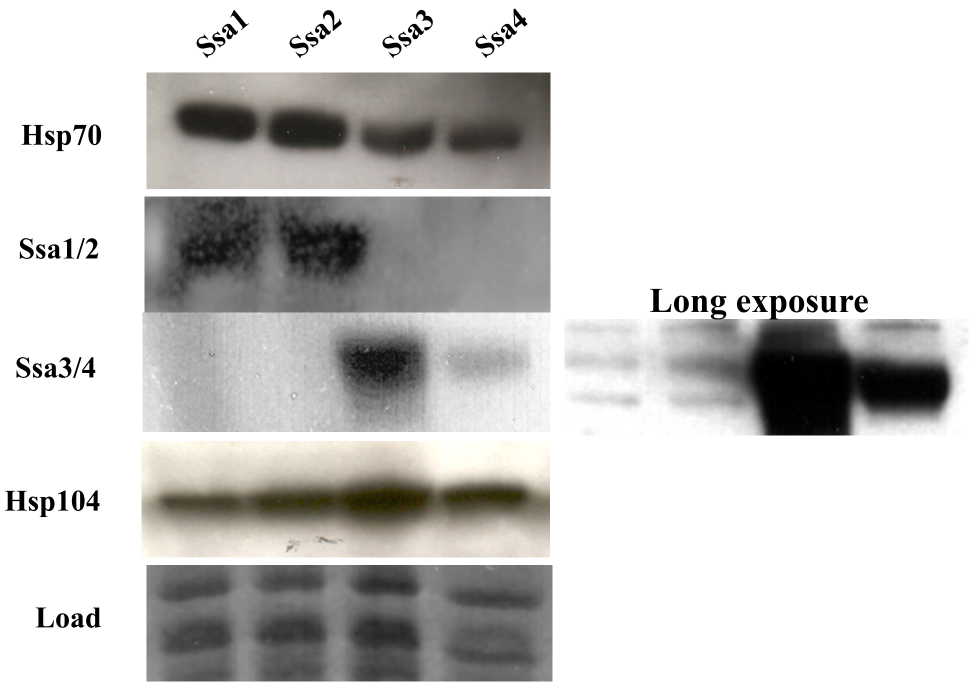

Figure S1: Expression of individual Hsp70-Ssa family members in yeast. To confirm expression of individual Ssa family members we used Western blotting with antibodies recognising Hsp70-Ssa (top panel), Ssa1 or Ssa2 only and Ssa3 or Ssa4 only. We also assessed Hsp104 expression in these cells. Ssa1/2 and Ssa3/4 specific antibodies were a gift from Elizabeth Craig. Hsp104 antibody was a gift from John Glover. Loading control is membrane stained with amido black.
