# Supplementary material for: Global transcript and phenotypic analysis of yeast cells expressing Ssa1, Ssa2, Ssa3 or Ssa4 as sole source of cytosolic Hsp70-Ssa chaperone activity
Source: BMC Genomics. 2014 Mar 14;15(1):194. doi: 10.1186/1471-2164-15-194 (PMC4022180; doi:10.1186/1471-2164-15-194)
Supplement: Supplementary file 3 — Additional file 3: Figure S2: Relative abundance of Hsp70 and Hsp104 in yeast cells expressing. individual Ssa’s. Western blot analysis was performed to examine the abundance of Hsp70 and Hsp104. Blots probed with anti-Hsp70 antibodies (SPA822, Cambridge Biosciences) were stripped and re-probed with anti-Hsp104 antibodies (gift from John Glover). Membrane was then stained by amido black as a loading and transfer control, are shown (Load). The Ssa strains are indicated on the top. (PDF 237 KB) [file 12864_2013_7032_MOESM3_ESM.pdf]

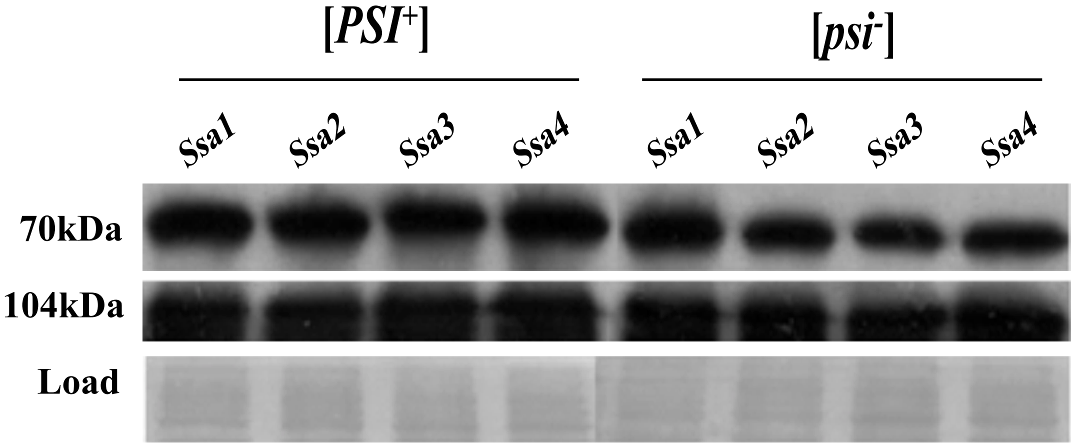

Figure S2: Relative abundance of Hsp70 and Hsp104 in yeast cells expressing individual Ssa's. Western blot analysis was performed to examine the abundance of Hsp70 and Hsp104. Blots probed with anti-Hsp70 antibodies (SPA822, Cambridge Biosciences) were stripped and re-probed with anti-Hsp104 antibodies (gift from John Glover). Membrane was then stained by amido black as a loading and transfer control, are shown (Load). The Ssa strains are indicated on the top.
