# Supplementary material for: Global transcript and phenotypic analysis of yeast cells expressing Ssa1, Ssa2, Ssa3 or Ssa4 as sole source of cytosolic Hsp70-Ssa chaperone activity
Source: BMC Genomics. 2014 Mar 14;15(1):194. doi: 10.1186/1471-2164-15-194 (PMC4022180; doi:10.1186/1471-2164-15-194)
Supplement: Supplementary file 5 — Additional file 5: Table S3: Comparative overview of fold change for microarray and qPCR of 25 genes. (PDF 61 KB) [file 12864_2013_7032_MOESM5_ESM.pdf]

Table S3: Comparative overview of fold change for microarray and qPCR of 25 genes

Gene name	Systematic name	Microarray			qPCR		
		Ssa2	Ssa3	Ssa4	Ssa2	Ssa3	Ssa4
CBP6	YBR120C	1.39	1.94	2.14	1.71	1.65	2.24
-	YBR197C	0.44	0.62	0.60	0.75	0.86	0.88
ILV6	YCL009C	0.22	1.10	1.00	0.90	0.84	0.56
LYS20	YDL182W	0.26	1.64	2.46	1.09	0.97	2.21
GPI8	YDR331W	1.12	0.16	0.97	1.21	0.81	0.94
HXT7	YDR342C	2.76	6.15	1.51	2.12	1.79	3.37
ARO9	YHR137W	0.83	0.50	0.11	0.94	0.39	0.10
MIF2	YKL089W	1.12	0.08	1.13	1.34	0.72	0.61
YOX1	YML027W	1.30	0.56	0.23	0.50	0.41	0.39
CLN1	YMR199W	2.48	2.24	0.81	1.22	0.68	0.20
SCJ1	YMR214W	1.29	0.60	0.08	1.16	0.50	0.45
ADH2	YMR303C	0.21	0.38	0.04	0.14	0.22	0.02
HUF1	YNL108C	0.18	1.18	1.17	1.08	0.90	1.19
UBP10	YNL186W	1.07	0.28	1.03	0.97	0.78	0.78
TOS6	YNL300W	1.39	1.11	0.21	1.57	0.71	0.17
-	YNR014W	3.38	2.44	10.37	2.77	2.33	7.24
-	YOR387C	2.96	0.54	0.26	2.21	0.55	0.42
OYE3	YPL171C	3.31	3.31	12.45	6.11	6.00	32.87
CLN2	YPL256C	0.90	1.48	0.47	1.03	1.00	0.45
HXT1	YHR094C	2.69	3.05	16.90	0.55	0.65	1.07
MRPL6	YHR147C	9.81	13.31	11.98	2.13	2.03	2.18
ATG8	YBL078C	6.69	5.76	10.23	0.89	0.85	1.16
HSP31	YDR533C	6.64	5.55	14.08	1.48	1.73	2.13
VMA13	YPR036W	0.97	1.00	1.02	0.78	0.67	1.04
MSS116	YDR194C	5.70	3.09	4.83	1.18	0.98	1.62
